# Evaluating the efficiency of ELISA, monoplex and multiplex probe‐based real‐time reverse‐transcription PCR assays in the detection of SARS‐CoV‐2 (COVID‐19) and influenza A and B viruses: A cross‐sectional study

**DOI:** 10.1002/hsr2.2140

**Published:** 2024-06-24

**Authors:** Mehrdad Mosadegh, Shirin Jalili, Mohammad Reza Pourmand, Yousef Erfani, Mohammad Panji

**Affiliations:** ^1^ Department of Pathobiology, School of Public Health Tehran University of Medical Sciences Tehran Iran; ^2^ Institute of Police Equipment and Technologies Policing Sciences and Social Studies Research Institute Tehran Iran; ^3^ Department of Medical Laboratory Sciences, School of Allied Medical Sciences Tehran University of Medical Sciences Tehran Iran; ^4^ Research Center for Life and Health Sciences and Biotechnology for the Police, Directorate of Health, Rescue and Treatment Police Headquarter Tehran Iran

**Keywords:** COVID‐19, ELISA, influenza A, influenza B, monoplex RT‐PCR, multiplex rRT‐PCR, SARS‐CoV‐2

## Abstract

**Background and Aims:**

The current study aimed to evaluate the efficiency of Enzyme‐linked immunosorbent assay (ELISA) assay and monoplex and multiplex real‐time reverse‐transcription PCR (rRT‐PCR) in the detection of severe acute respiratory syndrome coronavirus 2 (SARS‐CoV‐2) and influenza A and B viruses (Flu A and Flu B).

**Methods:**

The SARS‐CoV‐2 ‐specific IgG and IgM antibodies, as well as, Flu A (H1N1 and H3N2 serotypes) and Flu B virus antibodies were determined by ELISA assay. The one‐step qRT‐PCR method was used to detect the SARS‐CoV‐2 in nasopharyngeal swab samples. Furthermore, the presence of Flu A and B viruses was evaluated using probe‐based RT‐PCR. Simultaneous detection of SARS‐CoV‐2, Flu A and B viruses was performed by multiplex rRT‐PCR assay.

**Results:**

SARS CoV‐2 IgM and IgG antibodies were detected in 33.3% and 58.3% of patients, respectively. In contrast, the SARS CoV‐2 genome was detected in 50% of patients using the one‐step monoplex RT‐PCR assay. Flu A serotypes H1N1 and H3N2 were found in 16.7% and 8.3% of patients. Probe‐based RT‐PCR revealed that 39.3% of patients were positive for the Flu A virus. Multiplex rRT‐PCR detect the SARS‐CoV‐2, Flu A, and Flu B in 50%, 39.3%, and 19% of samples, respectively. The sensitivity and specificity of multiplex rRT‐PCR assay in comparison to monoplex RT‐PCR were 100% and 55%, respectively. Coinfection with SARS‐CoV‐2, Flu A, and Flu B viruses was found in 9.5% of patients.

**Conclusion:**

Multiplex rRT‐PCR can be used as a repaid, cost‐effective and suitable tool for molecular surveillance of SARS‐CoV‐2 and Flu A/B viruses.

## 1 INTRODUCTION

The novel coronavirus severe acute respiratory syndrome coronavirus 2 (SARS‐CoV‐2) emerged in December 2019 and spread globally. This virus is responsible for the COVID‐19 pandemic and is considered a serious global public health threat.^1^ Clinical manifestations of the novel coronavirus SARS‐CoV‐2 is varied from mild illness to severe infections such as acute respiratory distress syndrome (ARDS).^2^

According to the data registered in the World Health Organization (WHO) online site, as of March 10, 2024, 774,889,074 million confirmed cases and 7,038,623 deaths have been identified worldwide. https://covid19.who.int/. In Iran, from 3 January 2020 to 26 March 2024, 7,627,186 confirmed cases (103,142 active cases) of COVID‐19 and 146,811 deaths were reported. https://www.worldometers.info/coronavirus/country/iran/. Moreover, as of 18 February 2023, a total of 157,785,811 vaccine doses have been used in Iran. https://covid19.who.int/region/emro/country/ir.

Flu A and Flu B are respiratory viruses and outbreaks of these pathogens are seen every year in autumn and winter (colder months of the year).^3^ In most cases, the mode of transmission, management, and clinical presentations of the Flu virus and coronavirus SARS‐CoV‐2 are similar.^4^ Abnormalities caused by the Flu virus is varied from uncomplicated upper respiratory tract disease to complicated illnesses with severe viral pneumonia.^3^ It is well known that Flu disease is responsible for 650,000 deaths annually.^5^ In general, children and individuals aged > 65 years are the susceptible groups to Flu infections.^4^

Healthcare systems are likely to face overlapping outbreaks of SARS‐CoV‐2 and influenza. Flu‐like symptoms are common in Influenza and SARS‐CoV‐2, which makes it difficult to differentiate them solely on clinical presentation.^6^

Up to 25% of the human population worldwide can be infected by seasonal outbreaks caused by influenza viruses. Three mechanisms are available for influenza viruses to spread from person to person: aerosols, droplets, and contact transmission.^7^ Influenza can be classified as an asymptomatic infection or a fulminant illness in terms of clinical presentation and systemic symptoms. Fever, chills, headache, myalgia, malaise, and anorexia are all typical symptoms of influenza. Dry cough, pharyngeal pain, nasal discharge, and obstruction are respiratory symptoms that are present at the onset of the disease.^6^ In the general population, COVID‐19 is associated with fever, cough, dyspnea, malaise, fatigue, and sputum.^8^

As the mode of transmission, clinical manifestations, and epidemiological aspects of the influenza virus and COVID‐19 have many shared features, the co‐colonization of Flu and COVID‐19 can complicate the precise detection of SARS‐CoV‐2.^9^, ^10^ The United States Centers for Disease Control and Prevention (CDC) and WHO encourage researchers and scientists to test samples of patients infected with COVID‐19 infection for the Flu virus.^11^ Therefore, the use of sensitive and specific methods to surveillance and optimal management of both respiratory viruses is important.

To date, several molecular and serological assays have been introduced to the detection of SARS‐CoV‐2 and Flu viruses.^12^, ^13^, ^14^ Rapid antigen test, Rapid antibody test, RT‐PCR, Loop‐mediated isothermal amplification, CRISPR, and Next‐generation sequencing are SARS‐CoV‐2 diagnostic assays.^15^, ^16^ The diagnosis of both SARS‐CoV‐2 and influenza infections is still reliant on RT‐PCR as the gold standard.^17^

In the present study, we aimed to describe the clinical manifestation and laboratory biomarkers of patients hospitalized with COVID‐19 and Flu infections. Moreover, we evaluated the efficiency of Enzyme‐linked immunosorbent assay (ELISA) as a main serology test and monoplex and multiplex rRT‐PCR as the gold standard assays in the detection of SARS‐CoV‐2 and Flu A and Flu B viruses.

## 2 MATERIALS AND METHODS

### 2.1 Study design, sampling, and data collection

The present study was performed from May 2021 to August 2022 and was approved by the Ethics committee of the Tehran University of Medical Sciences (IR.TUMS.SPH.REC.1401.110). A total of 84 patients with different clinical symptoms of respiratory tract infections were included in the present study. In the first step, the research aims were explained to all participants, written informed consent was provided, and all included patients were signed. In the next step, a questionnaire was designed and all sociodemographic and clinical data including gender, age, fever, cough, and sore throat were recorded. Moreover, the results of laboratory biomarkers including white blood cell (WBC) count, neutrophil percentage, neutrophil count, lymphocyte percentage, and lymphocyte count were gathered. After data collection, sampling was performed on each patient. For this purpose, 5 milliliters of blood sample plus a nasopharyngeal swab (flock swabs, Copan) was taken from each patient. All blood samples were centrifuged at 10,000 rpm for 10 min and serum samples were stored at −20°C until further analysis. Moreover, all swab samples were suspended in 10 mL of sterile PBS (phosphate‐buffered saline, 0.1 M, pH 7.2; Sigma‐Aldrich®. Supplier: Merck) and stored until further analyses.

### 2.2 Determination of SARS‐CoV‐2 ‐specific IgG antibodies by ELISA

All serum samples were evaluated in duplicate for IgG antibodies against SARS‐CoV‐2 N antigen (Nucleocapsid protein) using the SARS‐CoV‐2 IgG ELISA Kit (Pishtaz Teb). The test was performed according to the manufacturer's instructions. In the first step, we diluted serum samples 1:101 with sample diluent (10 µL of the sample with 1000 µL of sample diluent). For IgG antibody detection, 100 μL of each control serum and diluted serum samples were added into appropriate wells. Two first wells were used as the blank and the next two wells as the negative control. Positive controls were used as duplicates. In the next step, all wells were sealed with cardboard sealer tightly and plates were incubated at 37°C for 30 min. After the incubation period, the contents of the wells were removed by flicking plate contents into a waste container and all wells were washed with 300 μL of working wash solution (5 times). To remove all residual water droplets, we struck the wells sharply onto absorbent paper. Following the washing step, 100 µL of antihuman IgG‐HRP conjugate was added into the wells and all wells were sealed with cardboard sealer tightly and plates were incubated at 37°C for 30 min. The contents of the wells were removed by flicking plate contents and all wells were washed with 300 μL of working wash solution (5 times). we struck the wells sharply onto absorbent paper and 100 μL of the chromogen‐substrate solution was immediately added to each well. Microplate wells were incubated at room temperature and in the darkness for 15 min to develop color. Finally, 100 μL of stop solution was added to the wells to stop the reaction and the absorbance of all wells was read with an ELISA reader at 450 nm.

### 2.3 Determination of SARS‐CoV‐2 ‐specific IgM antibodies by ELISA

Serum samples were tested in duplicate for specific IgM antibodies against N (Nucleocapsid protein) and S (spike protein) SARS‐CoV‐2 antigens using the SARS‐CoV‐2 IgM Capture ELISA Kit (Pishtaz Teb, Iran). According to the manufacturer's instructions, we diluted serum samples 1:101 with sample diluent (10 µL of the sample with 1000 µL of sample diluent). Briefly, for the IgM antibody detection, 100 μL of sample diluent was added to all selected wells. Similar to the determination of IgG antibody, positive controls were used as duplicates. Two first wells were used as the blank and the next two wells as the negative control. Then 50 μL of positive control, negative positive control, and samples (in duplicate) were added into the corresponding wells. The contents of the wells were mixed by gently shaking for 30 s and the wells were sealed with cardboard sealer and incubated for 30 min at 37°C. After 30 min, the contents of the wells were removed by flicking plate contents into a waste container and all wells were washed 5 times with 300 μL of working wash solution. After the washing step, 100 μL of working conjugate solution was added to all wells except the blanks. The wells were covered with a cardboard sealer tightly and incubated for 30 min at 37°C. The contents of the wells were removed and all wells were washed 5 times with 300 μL of working wash solution and 100 µL of chromogen‐substrate solution was added to each well.

The microplate wells were incubated at room temperature for 15 min and shielded from light to develop colour. At the final step, 100 μL of stop solution was added to the wells, and the absorbance of each well was read with an ELISA reader at 450 nm. All of the steps and determining the cut‐off amount were performed based on the manufacturer's instructions.

### 2.4 Detection of Flu A and Flu B viruses using ELISA assay

A solid‐phase sandwich ELISA kit developed and provided by Xiamen University, China was applied to the determination of specific IgG antibodies against the Flu A (H1N1) virus HA (Hemagglutinin) protein. The assay and determining the cut‐off amount were performed according to the standard ELISA protocol and the manufacturer's instructions. In brief, 100 mL of samples in a sample dilution buffer was added to the wells and the wells were sealed with cardboard sealer and incubated for 2 h at 37°C. After incubation time, plates were washed five times with 300 μL of working wash solution and then a horseradish peroxidase‐conjugated rabbit anti‐hemagglutinin polyclonal antibody was added to each well. Plates were incubated at 37°C for 30 min and all wells were washed five times with 300 μL of working wash solution. In the next step, 100 µL of tetramethylbenzidine substrate solution was added to each well. Finally, a stop solution was added to the wells, and the optical absorbance of the wells was read with an ELISA reader at 450 nm within 5 min. The determination of specific IgG antibodies against the Flu A (H3N2) virus was performed according to the previously published study performed by Luo et al.^18^ Moreover, the seroprevalence of specific IgG antibody against Flu B was determined based on the previously published study performed by Bishai et al.^19^

### 2.5 Detection of SARS‐CoV‐2 using monoplex RT‐PCR

A quantitative RT‐PCR system was used to detect SARS‐CoV‐2 in nasopharyngeal swab samples. For this purpose, the QIAamp Viral RNA Mini kit (Qiagen) was used to total RNA extraction from nasopharyngeal swab samples. The extraction process was performed according to the supplier's instructions. Extracted RNA was treated with 20 U of RQ1 RNAse‐free DNAse (Promega), and all RNA samples were suspended in diethylpyrocarbonate (DEPC)‐treated water (0.1% v/v). RevertAid First Strand complementary DNA (cDNA) Synthesis Kit (Fermentas) was used for cDNA synthesis. The cDNA synthesis was performed according to the previously published study by Azimi et al.^1^

The specific primer and probe sequences that target the RNA‐dependent RNA polymerase (*RdRp*) gene (nCoV_IP2 and nCoV_IP4) of SARS‐CoV‐2 are shown in Table 1. The volume of materials and reaction condition was as follows: 12 μL reaction mix ×2 (3 mM Mg), 0.5 μL MgSO_4_ (50 mM), 1 μL superscriptIII RT/Platinum Taq Mix, 0.5 μL of forward primer (10 mM), 0.5 μL of reverse primer (10 mM), 0.5 μL of specific probes (10 μM), 4 μL of RNA sample, and 6 μl sterile distilled water. The reaction was performed in a total volume of 25 μL. The following thermocycling condition was used in the amplification of the reaction: reverse transcription at 55°C for 20 min, denaturation at 95°C for 5 min, 45 cycles of denaturation at 95°C for 30 s, annealing at 58°C for the 30 s, and cooling at 40°C for 30 s.

**Table 1:** The sequences of primers and probes used in the monoplex RT‐PCR.

Primer & probes Sequences		
One‐Step monoplex Real time PCR (SARS‐CoV‐2)		
nCoV‐IP2	F	5′ ‐ ATGAGCTTAGTCCTGTTG‐3′
nCoV‐IP2	R	5′‐CTCCCTTTGTTGTGTTGT‐3′
Probe		5′‐Hex‐AGATGTCTTGTGCTGCCGGT A‐3′‐BHQ‐1
nCoV‐IP4	F	5′‐GGTAACTGGTATGATTTCG‐3′
nCoV‐IP4	R	5′‐CTGGTCAAGGTTAATATAGG‐3′
Probe		5′‐ Fam‐TCATACAAACCACGCCAGG‐3′‐BHQ‐1
Probe‐based real‐time PCR (influenza A and B viruses)		
M	F	5′ ‐ GGACTGCAGCGTAGACGCTT‐3′
	R	5′ ‐ CATCCTGTTGTATATGAGGCCCAT‐3′
Probe		5′‐FAM‐CTCAGTTATTCTGCTGGTGCACTTGCCA‐TAMRA‐3′
HA	F	5′‐AAATACGGTGGATTAAATAAAAGCAA‐3′
	R	5′‐CCAGCAATAGCTCCGAAGAAA‐3′
Probe		5′‐FAM‐ACCCATATTGGGCAATTTCCTATGGC‐TAMRA‐3′

Abbreviations: HA, hemagglutinin gene segment; M, matrix protein; SARS‐CoV‐2, severe acute respiratory syndrome coronavirus 2.

### 2.6 Detection of Flu A and Flu B viruses using monoplex RT‐PCR

To detection of Flu A and Flu B viruses in RNA samples extracted from nasopharyngeal swab samples, the probe‐based RT‐PCR with specific primers and probes was applied. The sequences of primers and probes are shown in Table 1. The RT‐PCR assay conditions and the volume of materials were set according to the previously published study by Elden et al.^20^ Briefly, amplification of the reaction was done using the Corbett 6000 Rotor‐Gene thermocycler (Corbett Research). The final volume of materials was 25 μL and consist of 12 μL of TaqMan Universal PCR master mix (TaqMan® Universal PCR Master Mix; Ampliqon), 0.5 μL of forward primer, 0.5 μL of reverse primer (Flu A and Flu B primers), 4 μL of cDNA, 0.5 μL of each probe, and 7.5 μL sterile distilled water. Finally, the RT‐PCR assay was done based on the following thermocycling condition: 50°C for 2 min, denaturation at 95°C for 10 min, 50 cycles of denaturation at 95°C for 20 s, annealing at 60°C for the 60 s.

### 2.7 Simultaneous detection of SARS‐CoV‐2, Flu A, and Flu B viruses

The primers and probes used in the simultaneous detection of SARS‐CoV‐2, Flu A, and Flu B viruses are listed in Table 2. The reaction was performed using a multiplex rRT‐PCR assay based on a published study by Ni et al.^21^ Briefly, the multiplex rRT‐PCR assay was performed under the following conditions: initial denaturation at 95°C for 1 min followed by 45 cycles at 95°C for 15 s, and annealing at 60°C for 45 s. Moreover, the improved concentrations were set in a total volume of 25 μL consisting of 12 μL of 2 x RT‐PCR solution, 2 μL of primer complex compound (10 mM), 1.5 μL of the enzyme complex, 2 μL of probe complex compound (10 μM), 4 μL of extracted RNA sample, and 3.5 μL sterile distilled water. RNase P (C1), labelled with BHQ‐1 and HEX fluorescent dye was used as an internal reference control.

**Table 2:** The sequences of primers and probes used in the multiplex RT‐PCR assay.

Primer and probes	Target	Sequences
hIAV		
F	Matrix protein (M)	GACCRATCCTGTCACCTCTGAC
R		AGGGCATTYTGGACAAAKCGTCTA
Probe		ROX‐TGCAGTCCTCGCTCACTGGGCACG‐BHQ2
hIBV		
F	Neuraminidase (NA)	GGGATAGARATGGTACATGATGGTG
R		TGTGACAGTGTCCCACAGCAG
Probe		CY5‐ACTTGGCACTCAGCRGCAACAGCC‐BHQ3
SARS‐CoV‐2 N gene		
F	Nucleocapsid (N)	CCCCAAGGTTTACCCAATAAT
R		GGTCTTCCTTGCCATGTTGA
Probe		VIC‐CTGCGTCTTGGTTCACCGCTCTCAC‐BHQ1
SARS‐CoV‐2 ORF1ab gene		
F	Open reading frame 1ab (ORF1ab)	CATACACTCGCTATGTCGATAAC
R		AGTGTCAATAAAGTCCAGTTGTTC
Probe		FAM‐CTTCTGTGGCCCTGATGGCTACCCTC‐BHQ1

Abbreviation: SARS‐CoV‐2, severe acute respiratory syndrome coronavirus 2.

### 2.8 Analyses and evaluation of sensitivity and specificity

All data about patients were included in SPSS software version 26 (SPSS Inc.) and analyzed using the *χ* ^2^ test. A *p* ≤ 0.05 was considered to be statistically significant. The specificity and sensitivity of multiplex rRT‐PCR assay in the simultaneous detection of SARS‐CoV‐2 and Flu A and Flu B viruses were assessed against monoplex RT‐PCR assay and serological tests. Moreover, positive and negative predictive values of the multiplex rRT‐PCR assay were calculated. All of the statistical tests used for the analyses.

## 3 RESULTS

### 3.1 Study population

A total of 84 patients with symptoms of respiratory tract infection were included, of which 43 (51.2%) and 41 (48.8%) patients were male and female, respectively. Demographic data and clinical symptoms of included patients are shown in Table 3. In general, 52.4% (*n* = 44/84) and 47.6% (*n* = 40/84) were in the age range of >50 years and ≤50 years, respectively. The frequency of clinical symptoms among patients was as follows: fever (81%; *n* = 68/84), cough (73.8%; *n* = 62/84), and sore throat (70.2%, *n* = 59/84). 79.8% (*n* = 67/84) of patients have a normal WBC count and in 63.1% (*n* = 53/84) of patients, the neutrophil count was low. Moreover, 89.3% (*n* = 75/84) of patients have a high lymphocyte count.

**Table 3:** Demographic information of patients and prevalence and clinical signs of SARS CoV‐2 infection in ELISA assay.

Characteristics	Total with characteristic *N* (%)	SARS‐CoV‐2 IgG	*p* Value	SARS‐CoV‐2 IgM	*p* Value	SARS‐CoV‐2 (Monoplex)	*p* Value
Gender							
Male	43 (51.2)	25 (58.1%)	0.971	16 (37.2%)	0.440	23 (53.5%)	0.513
Female	41 (48.8)	24 (58.5%)		12 (29.3%)		19 (46.3%)	
Age							
≤50	40 (47.6)	23 (57.5%)	0.883	11 (27.5%)	0.280	20 (50%)	1
>50	44 (52.4)	26 (59.1%)		17 (38.6%)		22 (50%)	
Fever							
Yes	68 (81)	41 (60.3%)	0.452	20 (29.4%)	0.116	33 (48.5%)	0.578
No	16 (19)	8 (50%)		8 (50%)		9 (56.3%)	
Sore throat							
Yes	59 (70.2)	34 (57.6%)	0.840	21 (35.6%)	0.500	34 (57.6)	0.032*
No	25 (29.8)	15 (60%)		7 (28%)		8 (32)	
Cough							
Yes	62 (73.8)	36 (58.1%)	0.933	18 (29%)	0.160	27 (43.5%)	0.047*
No	22 (26.2)	13 (59.1%)		10 (45.5%)		15 (68.2%)	
WBC count							
Normal	67 (79.8)	41 (61.2%)	0.291	24 (35.8%)	0.337	5 (29.4%)	0.057
Non normal	17 (20.2)	8 (47.1%)		4 (23.5%)		37 (55.2%)	
Neutrophil count							
High	31 (36.9)	16 (51.6%)	0.339	16 (51.6%)	0.262	12 (38.7%)	0.113
Low	53 (63.1)	33 (62.3%)		33 (62.3%)		30 (56.6%)	
Lymphocyte count							
High	75 (89.3)	44 (58.7%)	0.858	24 (32%)	0.454	35 (46.7)	0.070
Low	9 (10.7)	5 (55.6%)		4 (44.4%)		7 (77.8%)	

Abbreviations: SARS‐CoV‐2 WBC, white blood cell.

### 3.2 Serological finding

In general, the results of the ELISA assay were revealed in Table 3. 33.3% (*n* = 28/84) and 58.3% (*n* = 49/84) of patients were positive for SARS‐CoV‐2 IgM and IgG antibodies, respectively. There was no statistically significant difference between specific IgG and IgM SARS CoV‐2 antibodies and the age, gender, and clinical symptoms of patients (Table 3). Flu A serotypes H1N1 and H3N2 were found in 16.7% (*n* = 14/84) and 8.3% (*n* = 7/84) of patients. The prevalence of Flu A serotype H1N1 was higher among patients with cough, non‐normal WBC count, and lymphopenia. However, this difference was not statistically significant (Table 4). Specific IgM antibody against Flu B was detected in 13.1% (*n* = 11/84) of patients.

**Table 4:** Demographic information of patients and prevalence and clinical signs of Flu A and Flu B infections in ELISA and molecular assays.

Characteristics	Total with characteristic *N* (%)	Flu A IgG (H1N1)	*p* Value	Flu A IgG (H3N2)	*p* Value	Flu B IgM	*p* Value	Flu A (Monoplex)	*p* Value	Flu B (Monoplex)	*p* Value
*Gender*											
Male	43 (51.2)	8 (18.6%)	0.625	2 (4.7%)	0.211	8 (18.6%)	0.125	14 (32.6%)	0.196	10 (23.3%)	0.314
Female	41 (48.8)	6 (14.6%)		5 (12.2%)		3 (7.3%)		19 (46.3%)		6 (14.6%)	
*Age*											
≤50	40 (47.6)	6 (15%)	0.696	3 (7.5%)	0.792	7 (17.5%)	0.254	15 (37.5%)	0.825	9 (22.5%)	0.580
>50	44 (52.4)	8 (18.2%)		4 (9.1%)		4 (9.1%)		18 (40.9%)		7 (15.9%)	
*Fever*											
Yes	68 (81)	11 (16.2%)	0.804	7 (10.3%)	0.180	10 (14.7%)	0.367	27 (39.7%)	0.871	14 (20.6%)	0.459
No	16 (19)	3 (18.2%)		0 (0%)		1 (6.3%)		6 (37.5%)		2 (12.5%)	
*Sore throat*											
Yes	59 (70.2)	7 (11.9%)	0.070	5 (8.5%)	0.943	7 (11.9%)	0.607	19 (32.2%)	0.041*	11 (18.6%)	0.885
No	25 (29.8)	7 (28%)		2 (8%)		4 (16%)		14 (56%)		5 (20)	
*Cough*											
Yes	62 (73.8)	12 (19.4%)	0.267	7 (11.3%)	0.182	8 (12.9%)	0.930	27 (43.5%)	0.179	12 (19.4%)	0.904
No	22 (26.2)	2 (9.1%)		0 (0%)		3 (13.6%)		6 (27.3%)		4 (18.2%)	
*WBC count*											
Normal	67 (79.8)	10 (14.9%)	0.395	5 (7.5%)	0.567	10 (14.9%)	0.324	9 (52.9%)	0.197	3 (17.6%)	0.869
Non normal	17 (20.2)	4 (23.5%)		2 (11.8%)		1 (5.9%)		24 (35.8%)		13 (19.4%)	
*Neutrophil count*											
High	31 (36.9)	5 (16.1%)	0.919	4 (12.9%)	0.246	6 (19.4%)	0.193	14 (45.2%)	0.399	9 (29%)	0.075
Low	53 (63.1)	9 (17%)		3 (5.7%)		5 (9.4%)		19 (35.8%)		7 (13.2%)	
*Lymphocyte count*											
High	75 (89.3)	12 (16%)	0.636	7 (9.3%)	0.338	10 (13.3%)	0.852	30 (40)	0.696	14 (18.7%)	0.797
Low	9 (10.7)	2 (22.2%)		0 (0%)		1 (11.1%)		3 (33.3%)		2 (22.2%)	

Abbreviation: WBC, white blood cell.

### 3.3 Monoplex rRT‐PCR results

Results obtained from monoplex and multiplex rRT‐PCR methods are shown in Tables 3 and 4. SARS CoV‐2 genome was detected in 50% (*n* = 42/84) of patients using one‐step monoplex RT‐PCR. There was a statistically significant relationship between SARS CoV‐2 infection and sore throat (*p* = 0.032) and cough (*p* = 0.047). Results of probe‐based RT‐PCR revealed that 39.3% (*n* = 33/84) and 19% (*n* = 16/84) of patients were positive for Flu A and Flu B, respectively. It was interesting that the prevalence of Flu A infection in patients with a sore throat was low (*p* = 0.041).

### 3.4 Multiplex rRT‐PCR results

We used multiplex rRT‐PCR for the simultaneous detection of SARS‐CoV‐2, Flu A, and Flu B viruses. Similar to monoplex RT‐PCR, SARS‐CoV‐2, Flu A, and Flu B genome were found in 50% (*n* = 42/84), 39/3% (*n* = 33/84), and 19% (*n* = 16/84) of samples. Moreover, co‐infection with SARS‐CoV‐2, Flu A, and Flu B viruses was found in 9.5% (*n* = 8/84) of patients.

The specificity, sensitivity, positive predictive value, negative predictive value, and accuracy of multiplex rRT‐PCR assay in the detection of SARS‐CoV‐2, Flu A, and Flu B viruses in comparison to monoplex RT‐PCR and ELISA assay are summarized in Table 5. The sensitivity and negative predictive value of multiplex rRT‐PCR in the detection of the SARS‐CoV‐2 against monoplex RT‐PCR assay were 100%. Moreover, the specificity of multiplex rRT‐PCR in the detection of the SARS‐CoV‐2 in comparison to the monoplex RT‐PCR, SARS‐CoV‐2 IgM and IgG ELISA Kits were 55%, 67%, and 42%, respectively. In contrast, the specificity of multiplex rRT‐PCR in the detection of the Flu A in comparison to the monoplex RT‐PCR, Flu A/H1N1‐ELISA and Flu A/H3N2‐ELISA were 63%, 84%, and 91%, respectively.

**Table 5:** The specificity, sensitivity, PPV, NPV, and accuracy of multiplex real‐time PCR assay.

Efficiency	Covid‐19 (Monoplex real‐time PCR)	SARS‐CoV‐2‐IgM	SARS‐CoV‐2‐IgG
Multiplex real‐time PCR (SARS‐CoV‐2)		
Sensitivity	100%	37.5%	62.5%
Specificity	55%	67%	42%
PPV	19%	11%	10%
NPV	100%	91%	91%
Accuracy	59.5%	64%	44%

Efficiency	Flu A (Monoplex real‐time PCR)	Flu A/H1N1 ‐ ELISA	Flu A/H3N2 ‐ ELISA
Multiplex real‐time PCR (Flu A)		
Sensitivity	62.5%	25%	0%
Specificity	63%	84%	91%
PPV	15%	14%	0%
NPV	94%	91%	90%
Accuracy	63%	78%	82%

Efficiency	Flu B (Monoplex real‐time PCR)	Flu B (IgM) ‐ ELISA
Multiplex real‐time PCR (Flu B)		
Sensitivity	37.5%	37.5%
Specificity	83%	89%
PPV	19%	27%
NPV	93%	93%
Accuracy	78.5%	84.5%

Abbreviations: Flu A, Influenza A virus; Flu B, Influenza B virus; NPV, Negative predictive value; PPV, Positive predictive value.

## 4 DISCUSSION

Previously published studies revealed that co‐infection of SARS‐CoV‐2 with Flu A and Flu B viruses can occur in up to ~30% of patients.^2^ In most cases, the clinical symptoms of SARS‐CoV‐2 and Flu A and Flu B infections are very similar. Therefore, making a correct diagnosis and differentiation based on clinical signs alone is impossible.^4^, ^22^ Until now several serological and molecular‐based assays have been used to detect SARS‐CoV‐2 and Flu A and Flu B respiratory infections.^12^, ^13^, ^14^, ^18^, ^23^, ^24^ However, according to the high rate of co‐infection and high similarity in clinical signs between SARS‐CoV‐2 with Flu A and Flu B infections, as well as, to reduce the costs of patients and hospitals, it is necessary to use a method that simultaneously identifies these pathogens.

In the present study, we followed several aims including) determining the prevalence of SARS‐CoV‐2 IgG and IgM antibodies by ELISA assay, (2) determining the prevalence of Flu A (H1N1 and H3N2 serotypes) and Flu B by ELISA assay, (3) determine the prevalence of SARS‐CoV‐2, Flu A and Flu B by monoplex RT‐PCR assay, (4) the simultaneous detection of SARS‐CoV‐2, Flu A and Flu B by multiplex rRT‐PCR assay, (5) determine the sensitivity and specificity multiplex rRT‐PCR assay in simultaneously detection of SARS‐CoV‐2, Flu A and Flu B viruses.

Results showed that SARS CoV‐2 IgM and IgG antibodies were detected in 33.3% and 58.3% of patients, respectively. In contrast, the SARS CoV‐2 genome was detected in 50% of patients using the one‐step monoplex RT‐PCR assay. The presence of mild or asymptomatic patients, high contagiousness of the virus, similarity in clinical signs with other respiratory infections, the existence of many susceptible groups against the virus, and the development of severe disease in susceptible cases makes the SARS‐CoV‐2 a main pathogen and these aspects can affect the pandemic spread of this virus.^21^, ^25^

Flu A serotypes H1N1 and H3N2 were found in 16.7% and 8.3% of patients using ELISA assay. However, results of probe‐based RT‐PCR revealed that 39.3% of patients were positive for the Flu A virus. Specific IgM antibody against the Flu B virus was detected in 13.1% of patients. In contrast, probe‐based RT‐PCR showed that the prevalence of the Flu B virus was 19%. Antibody serology tests was useful in epidemiological studies.^24^ However, nucleic acid‐based tests such as RT‐PCR are very appropriate for the detection of infections in patients. Nucleic acid‐based tests are considered the gold standard assays in the detection of SARS‐CoV‐2, Flu A and Flu B.^9^, ^11^, ^26^

The sensitivity and specificity of multiplex rRT‐PCR assay in the detection of SARS CoV‐2 virus in comparison to monoplex RT‐PCR was 100% and 55%, respectively. Similar to monoplex RT‐PCR, multiplex rRT‐PCR detected SARS‐CoV‐2, Flu A, and Flu B in 50%, 39.3%, and 19% of samples, respectively. Multiplex rRT‐PCR assay is recommended by the Centers for Disease Control and Prevention (CDC) as a highly perfect and accurate nucleic acid‐based diagnostic assay to simultaneously detect and differentiate between SARS‐CoV‐2, Flu A, and Flu B in patients with upper or lower acute respiratory infections.^26^ Compared to the monoplex rRT‐PCR, multiplex rRT‐PCR is a rapid and cost‐effective assay. This assay can identify several targets in one reaction and is easy to manage.^14^ Moreover, the use of multiplex rRT‐PCR decreases the contamination rate and pipetting error and reduces the freeze‐thaw cycles of samples and materials.^12^

Co‐infection with SARS‐CoV‐2, Flu A, and Flu B viruses was found in 9.5% of patients. Although coinfection between SARS‐CoV‐2 and Flu A and Flu B was reported in different published studies,^1^, ^27^, ^28^, ^29^ however, so far, the exact mechanism and viral kinetics of SARS‐CoV‐2 coinfections are not determined. This is due to the similarity in clinical signs between circulating respiratory pathogens such as SARS‐CoV‐2 and Flu A (serotype H1N1).^22^, ^30^ However, it is presumed that coinfection between SARS‐CoV‐2 and other viruses is associated with the immunological status of each patient and the mechanisms involved in viral interference.^3^, ^28^, ^31^ In general, coinfection between viruses is related to disease severity and leads to an increase in the duration of hospitalization in intensive care units and the development of ARDS. In most cases, coinfection is related to high‐case fatality.^28^, ^32^

The present study was a research with a budget limitation and have several limitations. The main limitation of the present study was the low number of samples. The exact surveillance of coinfection requires a high sample size. Moreover, although we designed a questionnaire and collect clinical and demographic data of patients, however, some required data such as the frequency of underlying diseases among included patients, duration of hospitalization, and a history of antibiotic usage were not available.

## 5 CONCLUSION

In conclusion, our analyses revealed that results obtained from multiplex rRT‐PCR are similar to results of monoplex RT‐PCR assay. Moreover, in comparison to monoplex RT‐PCR, the sensitivity of multiplex rRT‐PCR in the detection of SARS‐CoV‐2 was 100%. Therefore, multiplex rRT‐PCR can be used as a repaid, cost‐effective and suitable tool for molecular surveillance of SARS‐CoV‐2 and Flu A/B viruses.

## AUTHOR CONTRIBUTIONS

Mehrdad Mosadegh, Shirin Jalili, and Mohammad Panji contributed to the conceptualization of the study, and drafting and revising the manuscript; Mohammad Reza Pourmand, Mehrdad Mosadegh, Yousef Erfani, and Mohammad Panji contributed to sample acquisition, data generation, data analysis and data interpretation.

## CONFLICT OF INTEREST STATEMENT

The authors declare no conflict of interest.

## ETHICS STATEMENT

All authors have read and approved the final version of the manuscript. Dr. Shirin Jalili had full access to all of the data in this study and takes complete responsibility for the integrity of the data and the accuracy of the data analysis.

## TRANSPARENCY STATEMENT

The lead author Shirin Jalili affirms that this manuscript is an honest, accurate, and transparent account of the study being reported; that no important aspects of the study have been omitted; and that any discrepancies from the study as planned (and, if relevant, registered) have been explained.

## Data Availability

All relevant data are within the manuscript.
